# Polysomnography in otorhinolaryngology (1995–2025): Bibliometric analysis and database comparison

**DOI:** 10.1097/MD.0000000000049749

**Published:** 2026-07-10

**Authors:** Ömer Faruk Zengin, Gözde Salihoglu Zengin

**Affiliations:** a Department of Otorhinolaryngology, Ordu University Training and Research Hospital, Ordu, Turkey; b Department of Psychiatry, Ordu University Training and Research Hospital, Ordu, Turkey.

**Keywords:** bibliometrics, citation analysis, otorhinolaryngology, polysomnography

## Abstract

**Background:**

Polysomnography (PSG) is essential for diagnosing sleep-related disorders in otorhinolaryngology. Despite its clinical importance, to our knowledge, no comprehensive bibliometric study has examined PSG research in this area. This study aimed to provide a 30-year bibliometric analysis (1995–2025) of articles related to PSG in otorhinolaryngology journals, assessing publication trends, author, journal, and institution contributions, keyword dynamics, and cross-database citation comparability, which is important as citation metrics vary across databases.

**Methods:**

Original articles were retrieved from Web of Science (WoS) and afterwards from PubMed for each journal in the WoS otorhinolaryngology category using the keywords polysomnography, PSG, and sleep monitoring; duplicates were removed using titles and digital object identifiers. Bibliographic data were standardized and analyzed using descriptive statistics, correlation tests, and Bland–Altman plots. We performed bibliometric mapping (co-citation and keyword co-occurrence) using VOSviewer and forecasted publication trends using simple exponential smoothing.

**Results:**

A total of 1809 articles were included. Publication output increased significantly after 2010, with a projection of 121 articles by 2030. The USA, Türkiye, China, South Korea, and Taiwan were the leading contributors, and Laryngoscope and Otolaryngology–Head and Neck Surgery were the most influential journals. Keyword analysis revealed a recent focus on drug-induced sleep endoscopy, hypoglossal nerve stimulation, and robotic surgery. Although citation counts across databases were highly correlated with WoS (*r* = 0.967–0.983), Bland–Altman analysis revealed that WoS tended to yield lower citations, particularly for highly cited articles.

**Conclusion:**

PSG research in otorhinolaryngology has experienced sustained growth over the past 15 years, driven by strong international collaboration. In addition, this study outlines the trends in PSG research and emphasizes the impact of database selection on bibliometric outcomes.

## 1. Introduction

Polysomnography (PSG) remains the reference diagnostic method for sleep evaluation and detection of sleep-related disorders, such as obstructive sleep apnea (OSA), parasomnias, and central sleep disturbances. It involves continuous and concurrent monitoring of multiple physiological signals during sleep. Over time, its parameters have expanded to include electroencephalography, electrooculography, electromyography, respiratory and cardiac channels, body position, capnography, and video monitoring.^[1,2]^ Although the use of home sleep apnea testing has increased in recent years, PSG performed in a laboratory remains the reference standard in clinical practice, particularly in otorhinolaryngology.^[3]^

The clinical importance of PSG lies in its role in the diagnosis of OSA, the guidance it provides in treatment adjustments, and its application in postoperative or long-term follow-up.^[3]^

The standardization of PSG scoring has evolved from the Rechtschaffen and Kales manual to the American Academy of Sleep Medicine criteria. Updates to the latter have improved the objectivity of respiratory event scoring and interrater reliability.^[2,4]^

Technological advances – from analog to digital systems and expanded electroencephalography montages – have further refined data quality and interpretability.^[1]^

This clinical importance is reflected by the notable increase in PSG publications over the past 3 decades. Despite this expansion, detailed bibliometric investigations tracking long-term publication activity, citation performance, and international collaboration remain lacking, particularly in otorhinolaryngology literature. Originally developed in the field of library and information science, bibliometric analysis has become an increasingly valuable tool in medicine. It offers a quantitative method of evaluating research productivity, highlighting influential contributors and identifying emerging trends.^[5]^

Recent bibliometric studies in sleep medicine have explored topics such as OSA and anesthesia,^[6]^ inflammation in OSA,^[7]^ neuroimaging in OSA,^[8]^ and wearable devices in sleep science,^[9]^ highlighting the utility of this approach. To our knowledge, no study has systematically examined the bibliometric landscape of PSG research in otorhinolaryngology. Moreover, cross-database citation comparisons are absent from PSG research.

This study aimed to conduct a comprehensive bibliometric analysis of PSG-related articles in otorhinolaryngology journals indexed in Web of Science (WoS) over the past 30 years (1995–2025). Publication trends, contributions of authors, countries, and institutions, citation comparisons across WoS, Google Scholar, Crossref, and OpenAlex, and keyword dynamics were analyzed. This study combines field-specific insights with a methodological perspective to highlight global developments in PSG research and contribute to the broader discussion on the comparability of bibliometric databases.

## 2. Materials and methods

### 2.1. Data sources and search strategy

This study was conducted using the Web of Science Core Collection (WoSCC; Clarivate Analytics) and PubMed databases, covering a 30-year period between 1995 and 2025. The following query was applied to WoSCC: TS = (polysomnography OR psg OR sleep monitoring) AND WC = (otorhinolaryngology) AND publication year = (1995–2025), with document type = (article). To achieve broader coverage, journals indexed in WoS under the otorhinolaryngology field were searched separately in PubMed using the following query: (polysomnography OR psg OR sleep monitoring) AND (“Arch Otolaryngol Head Neck Surg”[Journal]). The same search was repeated for each journal in this category.

The dataset also included articles that appeared in PubMed but not in WoS. Only original articles were considered, while reviews, case reports, meeting abstracts, editorials, letters, and corrections were excluded. Original articles were defined as research articles reporting primary data (e.g., clinical trials, cohort studies, case-control studies, and cross-sectional studies). In WoS, this classification was based on the “Document Type” field “Article.” In PubMed, we manually screened abstracts and publication types to confirm each record met the definition; any uncertainties were resolved by reviewer consensus. Data collection was conducted between August 1, 2025, and January 5, 2026.

For each eligible article, bibliographic information was exported into Microsoft Excel 2016, including journal title, article language, indexing status (science citation index expanded, emerging sources citation index), first author name, country, institutional affiliation, year of publication, citation counts, and keywords. Citation counts were collected and cross-verified using 4 databases: WoS, Google Scholar, Crossref, and OpenAlex.

**Figure 1.**
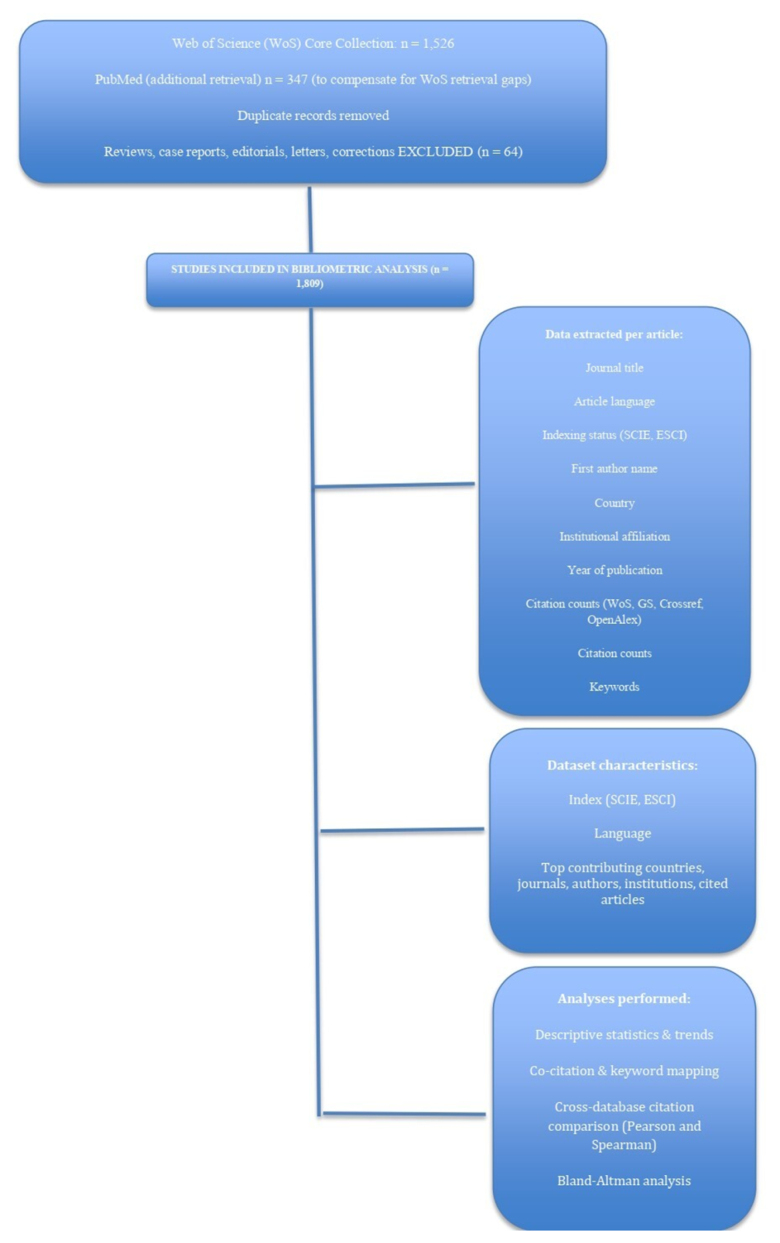
Flow diagram of the identification and selection process for polysomnography-related articles in otorhinolaryngology journals (1995–2025).

A systematic data-cleaning pipeline was implemented to ensure data integrity. First, additional articles from individual PubMed searches were cross-checked against the initial WoSCC dataset using titles and digital object identifier; all duplicate records were excluded. To guarantee thematic relevance, the abstracts of all retrieved articles were manually screened, and studies unrelated to PSG were removed. Author and institution names were manually standardized: spelling variations were corrected, and different affiliations belonging to the same institution were standardized. Second, keyword preprocessing was performed manually. All keywords were formatted using semicolon delimiters and corrected for typographical errors. Crucially, semantic synonym merging was intentionally avoided; spelling and linguistic variations (e.g., “apnea” vs “apnoea”) were preserved and quantified independently to accurately capture terminological preferences in the literature. The study selection process is depicted in Figure 1 as a flow diagram.

### 2.2. Statistical and bibliometric analysis

Descriptive statistics were performed using IBM SPSS Statistics for Windows, version 26.0 (IBM Corp.).

We analyzed publication trends and generated forecasts up to 2030 using a nonseasonal exponential triple smoothing model in Microsoft Excel 2016. The nonseasonal model was chosen because the data were aggregated annually, which naturally eliminated intrayear periodicity.

To evaluate consistency among citation databases, Pearson and Spearman correlation analyses were conducted and scatter plots (log-scaled) were generated to compare citation counts from WoS with those from Google Scholar, Crossref, and OpenAlex. Bland–Altman analysis was used to systematically examine the differences between the databases.

Comparative citation tables were prepared for the most-cited articles. Bibliometric mapping and network analyses, including keyword co-occurrence networks and co-citation analysis, were conducted using VOSviewer (version 1.6.20, Leiden University).

Two independent reviewers performed manual extraction and verification to minimize bias. Disagreements between the 2 reviewers were resolved through consensus.

This study was conducted in accordance with the BIBLIO guidelines.

## 3. Results

The WoS query initially identified 1526 publications, of which 1462 met the inclusion criteria. A separate search was performed in PubMed. Cross-database comparison using titles and digital object identifiers revealed retrieval gaps: the initial WoS search did not retrieve 347 articles present in PubMed, and PubMed did not retrieve 107 articles present in WoS. Manual verification confirmed that these articles were indexed in the respective databases but were not captured by the initial search.

After removing reviews, case reports, editorials, and biographical items, 1809 articles related to PSG were included. The majority (96.0%, n = 1738) were indexed in the science citation index expanded database, while only 71 (4.0%) were in the emerging sources citation index database.

The vast majority of articles were published in English (96.5%, n = 1745), with the remainder published in German (2.7%, n = 50) and Spanish (0.8%, n = 14).

### 3.1. Publication trends

**Figure 2.**
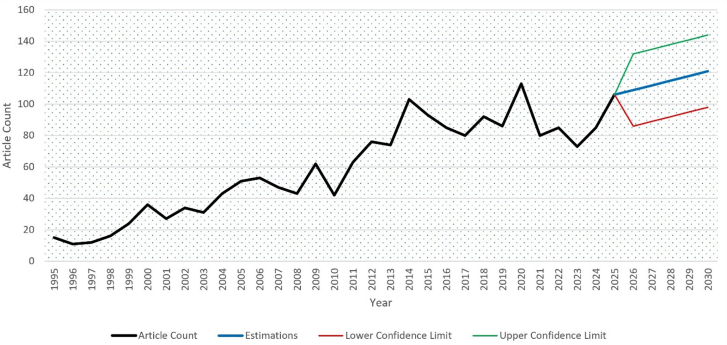
The line graph showing the historical publication trends in polysomnography (1995–2025) and projected article counts up to 2030.

Figure 2 depicts the historical trajectory and projected growth of PSG publications, as estimated using a nonseasonal exponential smoothing model. The results produced by the model predicted that the estimated number of articles related to PSG would be 109 (95% confidence interval [CI] = 86–132) in 2026 and would increase to 121 articles (95% CI = 98–144) by 2030.

The model adequacy was confirmed using formal error metrics. The model demonstrated high predictive accuracy, yielding a mean absolute scaled error of 1.13, a mean absolute error of 11.01, a root mean squared error of 14.06, and a symmetric mean absolute percentage error of 12.0%. These metrics indicate that the smoothed trend closely replicated the actual historical trajectory, whereas the 95% CIs provided stable bounds for future projections.

### 3.2. Contributions of country, journal, author, institution, and top-cited articles

**Figure 3.**
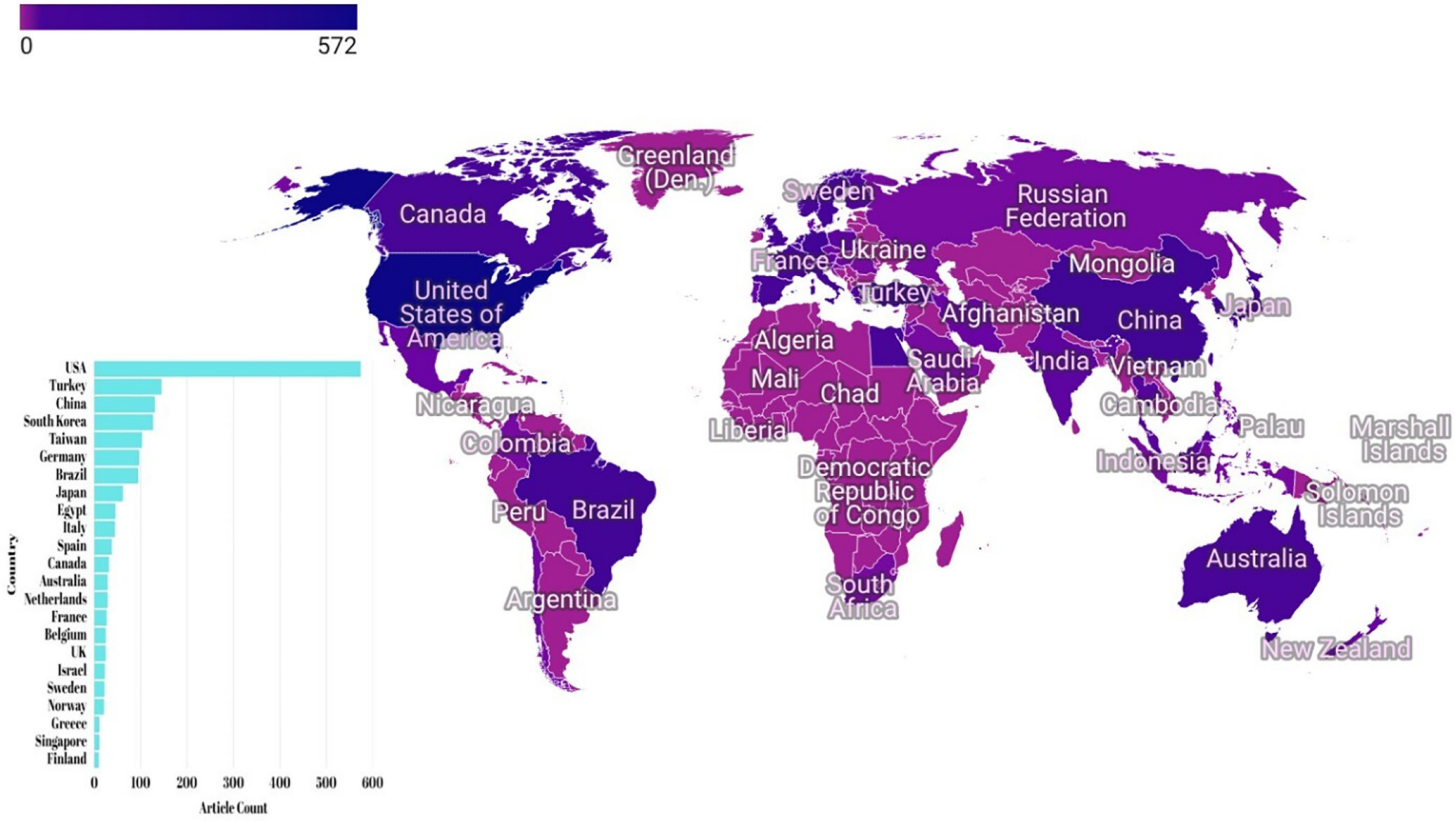
Bar graph of the top 23 countries contributing to the polysomnography literature.

Of the 49 countries that contributed publications on PSG, the 5 leading countries with over 100 articles were as follows: United States: 572 articles (31.6%, average citations per article: 28.63), Türkiye: 148 articles (8.2%, average citations per article: 13.09), China: 130 articles (7.2%, average citations per article: 11.02), South Korea: 127 articles (7.0%, average citations per article: 14.93), and Taiwan: 103 articles (5.7%, average citations per article: 20.98). Figure 3 depicts the top 23 countries contributing to PSG research (≥10 articles).

All publications on PSG appeared in 47 distinct journals. Table 1 presents the 29 journals (≥10 articles), together with their total number of WoS citations (number of co-citations [NC]) and mean NC per article.

**Table 1:** The leading 29 scientific journals with 10 or more articles on polysomnography.

Journal name	N*	C	AC	Journal name	N*	C	AC
Laryngoscope	272	9153	33.65	Clin Exp Otorhinolaryngol	26	275	10.57
Otolaryngol Head Neck Surg	270	7732	28.63	Laryngorhinootologie	21	140	6.66
Int J Pediatr Otorhinolaryngol	226	4270	18.89	ORL J Otorhinolaryngol Relat Spec	20	394	19.70
Eur Arch Otorhinolaryngol	221	3343	15.12	J Otolaryngol Head Neck Surg	20	313	15.65
Acta Otolaryngol	78	1464	18.76	Laryngoscope Investig Otolaryngol	15	50	3.33
Arch Otolaryngol Head Neck Surg	65	3673	56.50	Acta Otorrinolaringol Esp	14	85	6.07
Braz J Otorhinolaryngol	47	498	10.60	Acta Otorhinolaryngol Ital	13	150	11.54
Ann Otol Rhinol Laryngol	59	934	15.83	Egypt J Otolaryngol	13	17	1.30
Am J Otolaryngol	55	688	12.50	J Otolaryngol	12	186	15.5
Auris Nasus Larynx	48	383	7.97	B-ENT	10	74	7.40
Clin Otolaryngol	39	586	15.03	Int Arch Otorhinolaryngol	11	109	9.90
J Laryngol Otol	38	567	14.92	ENT Updates	11	7	0.63
JAMA Otolaryngol Head Neck Surg	38	882	23.21	Am J Rhinol Allergy	11	322	29.27
Ear Nose Throat J	14	79	5.64	Turk Arch Otorhinolaryngol	10	81	8.10
HNO	29	188	6.48				

AC = average citation per document, C = number of citations, N = number of articles.

* Only articles indexed in Web of Science were included in the calculation of average citations (retrieved on January 5, 2026).

Among the top contributors (≥20 articles) were Ron B. Mitchell (40 articles), Stacey L. Ishman (30 articles), Michael Friedman (29 articles), Jeong-Whun Kim (28 articles), and Hsin-Ching Lin (20 articles).

Leading institutional contributors (≥15 articles) to the PSG literature in ENT were: Chang Gung University (Taiwan, n = 56), University of São Paulo (Brazil, n = 44), University of Texas (USA, n = 41), Stanford University (USA, n = 31), University of Cincinnati (USA, n = 30), Capital Medical University (China, n = 28), Rush University (USA, n = 27), Seoul National University (South Korea, n = 27), Korea University (South Korea, n = 20), University of Pennsylvania (USA, n = 20), National Taiwan University (Taiwan, n = 20), Thomas Jefferson University (USA, n = 18), University of Antwerp (Belgium, n = 16), State University of New York (USA, n = 15), and University of Pittsburgh (USA, n = 15).

Table 2 presents the 40 most-cited articles (≥100 citations in WoS), listing the total and average annual citation counts.

**Table 2:** The first 40 high-impact articles with more than 100 citations based on the total number of citations on polysomnography.

Number	Article	First author	Journal	PY	TC	AC
1	Clinical predictors of obstructive sleep apnea	Friedman	Laryngoscope	1999	463	17.92
2	Quality of life for children with obstructive sleep apnea	Franco Jr	Otolaryngol Head Neck Surg	2000	401	16.04
3	Clinical staging for sleep-disordered breathing	Friedman	Otolaryngol Head Neck Surg	2002	354	15.43
4	Adenotonsillectomy for treatment of obstructive sleep-apnea in children	Suen	Arch Otolaryngol Head Neck Surg	1995	308	10.26
5	Pediatric sleep questionnaire: prediction of sleep apnea and outcomes	Chervin	Arch Otolaryngol Head Neck Surg	2007	275	15.27
6	Staging of obstructive sleep apnea/hypopnea syndrome: a guide to appropriate treatment	Friedman	Laryngoscope	2004	265	12.66
7	Adenotonsillectomy for obstructive sleep apnea in children: outcome evaluated by pre- and postoperative polysomnography	Mitchell	Laryngoscope	2007	248	13.83
8	Obstructive sleep apnea – should all children with Down syndrome be tested?	Shott	Arch Otolaryngol Head Neck Surg	2006	215	11.31
9	Three-year outcomes of cranial nerve stimulation for obstructive sleep apnea: the STAR trial	Woodson	Otolaryngol Head Neck Surg	2016	193	21.44
10	Outcome of adenotonsillectomy for obstructive sleep apnea in obese and normal-weight children	Mitchell	Otolaryngol Head Neck Surg	2007	192	10.66
11	Implanted upper airway stimulation device for obstructive sleep apnea	Van de Heyning	Laryngoscope	2012	184	14.23
12	Can history and physical examination reliably diagnose pediatric obstructive sleep apnea/hypopnea syndrome? A systematic review of the literature	Brietzke	Otolaryngol Head Neck Surg	2004	178	8.47
13	Effect of improved nasal breathing on obstructive sleep apnea	Friedman	Otolaryngol Head Neck Surg	2000	177	7.08
14	One hundred consecutive patients undergoing drug-induced sleep endoscopy: results and evaluation	Ravesloot	Laryngoscope	2011	176	12.57
15	Drug-induced sleep endoscopy in sleep-disordered breathing: report on 1249 cases	Vroegop	Laryngoscope	2014	169	15.36
16	Lateral pharyngoplasty: a new treatment for obstructive sleep apnea hypopnea syndrome	Cahali	Laryngoscope	2003	168	7.68
17	Interrater reliability of drug-induced sleep endoscopy	Kezirian	Arch Otolaryngol Head Neck Surg	2010	164	10.93
18	Radiofrequency tongue base reduction in sleep-disordered breathing: a pilot study	Powell	Otolaryngol Head Neck Surg	1999	156	6.00
19	The role of sleep position in obstructive sleep apnea syndrome	Richard	Eur Arch Otorhinolaryngol	2006	151	7.94
20	Sleep-disordered breathing in children: survey of current practice	Mitchell	Laryngoscope	2006	142	7.47
21	Surgery and obstructive sleep apnea: long-term clinical outcomes	Riley	Otolaryngol Head Neck Surg	2000	142	5.68
22	Snoring and obstructive sleep apnea in children - a 6-month follow-up study	Nieminen	Arch Otolaryngol Head Neck Surg	2000	134	5.36
23	Transoral robotic tongue base resection in obstructive sleep apnoea-hypopnoea syndrome: a preliminary report	Vicini	ORL J Otorhinolaryngol Relat Spec	2010	131	8.73
24	Surgery for obstructive sleep apnea: sleep endoscopy determinants of outcome	Koutsourelakis	Laryngoscope	2012	127	9.76
25	A comparison of Asian and white patients with obstructive sleep apnea syndrome	Li	Laryngoscope	1999	127	4.88
26	Effect of nasal surgery on sleep-related breathing disorders	Verse	Laryngoscope	2002	126	5.47
27	Obstructive sleep apnea is underrecognized and underdiagnosed in patients undergoing bariatric surgery	Ravesloot	Eur Arch Otorhinolaryngol	2012	123	9.61
28	Obstructive sleep apnea syndrome due to adenotonsillar hypertrophy in infants	Greenfeld	Int J Pediatr Otorhinolaryngol	2003	123	5.63
29	Direct hypoglossal nerve stimulation in obstructive sleep apnea	Eisele	Arch Otolaryngol Head Neck Surg	1997	123	4.39
30	Adenotonsillectomy and obstructive sleep apnea in children: a prospective survey	Guilleminault	Otolaryngol Head Neck Surg	2007	123	6.83
31	Accuracy of clinical evaluation in pediatric obstructive sleep apnea	Wang	Otolaryngol Head Neck Surg	1998	117	4.33
32	Association of systematic head and neck physical examination with severity of obstructive sleep apnea-hypopnea syndrome	Zonato	Laryngoscope	2003	112	5.09
33	Adenotonsillectomy for obstructive sleep apnea in obese children	Mitchell	Otolaryngol Head Neck Surg	2004	112	5.33
34	Polysomnography indexes are discordant with quality of life, symptoms, and reaction times in sleep apnea patients	Weaver	Otolaryngol Head Neck Surg	2005	111	5.55
35	Effects of adeno-tonsillectomy on polysomnography patterns in Down syndrome children with obstructive sleep apnea: a comparative study with children without Down syndrome	Shete	Int J Pediatr Otorhinolaryngol	2010	108	7.20
36	Sleep disordered breathing: surgical outcomes in prepubertal children	Guilleminault	Laryngoscope	2004	104	4.95
37	Reliability of the Muller maneuver and its association with sleep-disordered breathing	Terris	Laryngoscope	2000	102	4.08
38	Propofol-induced sleep: polysomnographic evaluation of patients with obstructive sleep apnea and controls	Rabelo	Otolaryngol Head Neck Surg	2009	101	6.31
39	Child behavior and quality of life in pediatric obstructive sleep apnea	Tran	Arch Otolaryngol Head Neck Surg	2005	101	5.05
40	Increased prevalence of obstructive sleep apnea in patients with cleft palate	Robison	Arch Otolaryngol Head Neck Surg	2011	100	7.14

AC = average citations per year, PY = publication year, TC = total citation.

### 3.3. Keyword and thematic evolution

The PSG dataset collectively used 2016 different keywords. Of these, 39 were used in at least 20 articles, as presented in Table 3.

**Table 3:** The most frequently used 39 keywords in published articles on polysomnography.

Keywords	NU	Keywords	NU
Obstructive sleep apnea	702	Quality of life	40
Polysomnography	374	OSAS	39
Snoring	171	Down syndrome	37
Sleep apnea	161	Apnea-hypopnea index	37
Adenotonsillectomy	109	Adenoidectomy	36
Obstructive sleep apnea syndrome	108	Sleep surgery	34
Tonsillectomy	95	Continuous positive airway pressure	33
Children	81	Pediatric obstructive sleep apnea	32
Uvulopalatopharyngoplasty	73	Sleep apnea syndromes	30
Drug-induced sleep endoscopy	69	Obstructive sleep apnea	29
Sleep-disordered breathing	67	Sleep endoscopy	27
Child	54	Epworth sleepiness scale	26
Obesity	54	Body mass index	26
Obstructive	50	Polysomnogram	26
OSA	49	Endoscopy	25
Sleep	48	Laryngomalacia	23
Pediatric	46	Nasal obstruction	23
Pediatrics	43	Apnea	21
Sleep disordered breathing	42	Diagnosis	21
Surgery	41		

NU = number of uses, OSA = obstructive sleep apnea.

**Figure 4.**
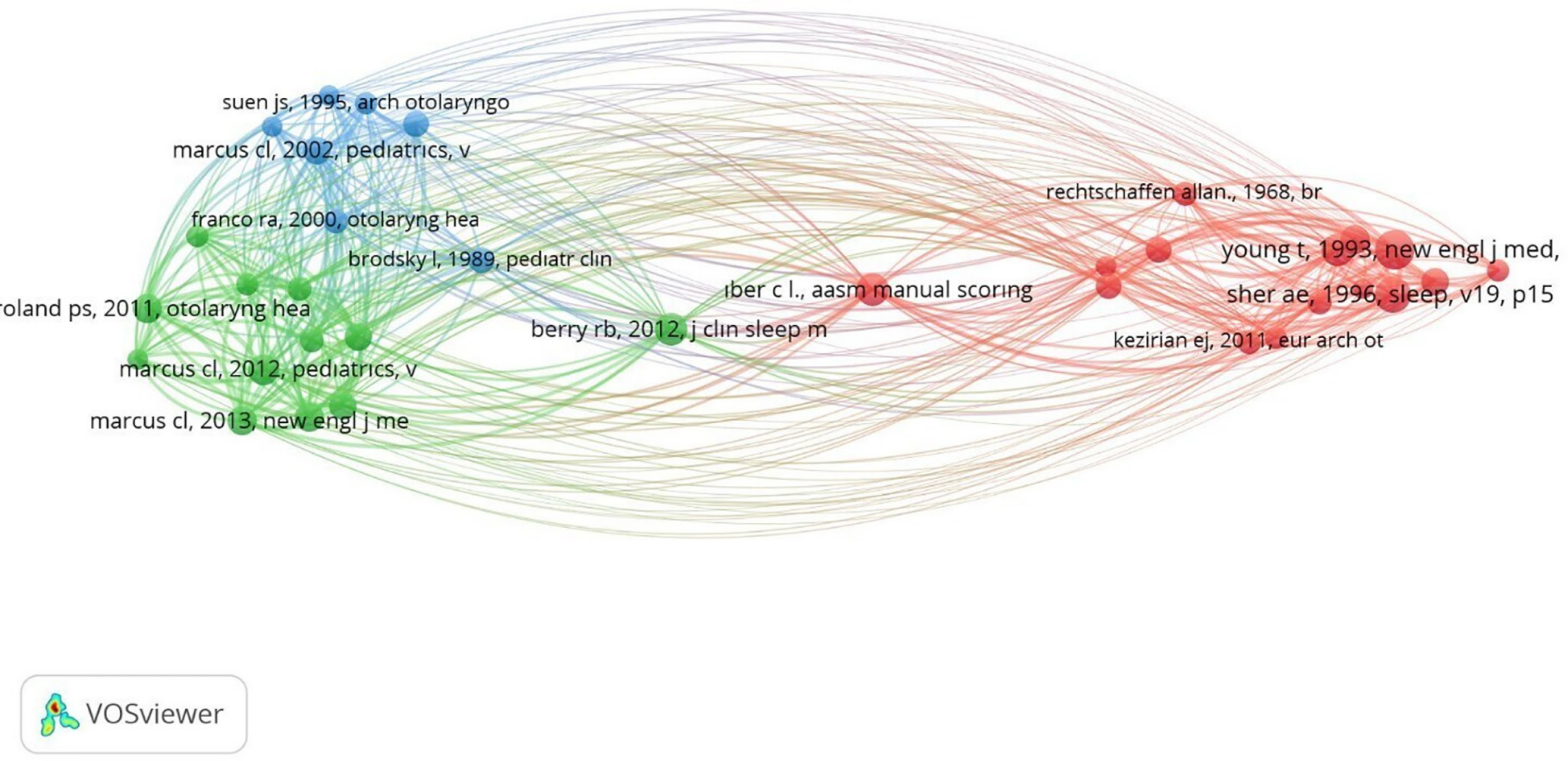
Network visualization of co-citation analysis in polysomnography research. Each node represents a cited reference and is labeled by the first author and publication year. The size of a node reflects the number of citations received, and the thickness of the connecting lines indicates the strength of co-citation relationships. The distance between nodes represents the degree of relatedness, with more closely positioned nodes indicating stronger associations. Different colors denote distinct co-citation clusters, representing major thematic areas within the polysomnography literature.

We performed co-citation mapping on cited references with a ≥50-citation threshold. Accordingly, 34 studies were identified as having been cited in more than 50 articles. Figure 4 depicts the co-citation network of PSG research articles. The reference lists of dataset articles contained 23,293 citations. The 7 most frequently co-cited studies (≥100 citations) included Young et al (NC: 183), Sher et al (NC: 156), Johns et al (NC: 122), Berry et al (NC: 122), Iber (NC: 119), Marcus et al (NC: 106), and Marcus et al (NC: 100).

**Figure 5.**
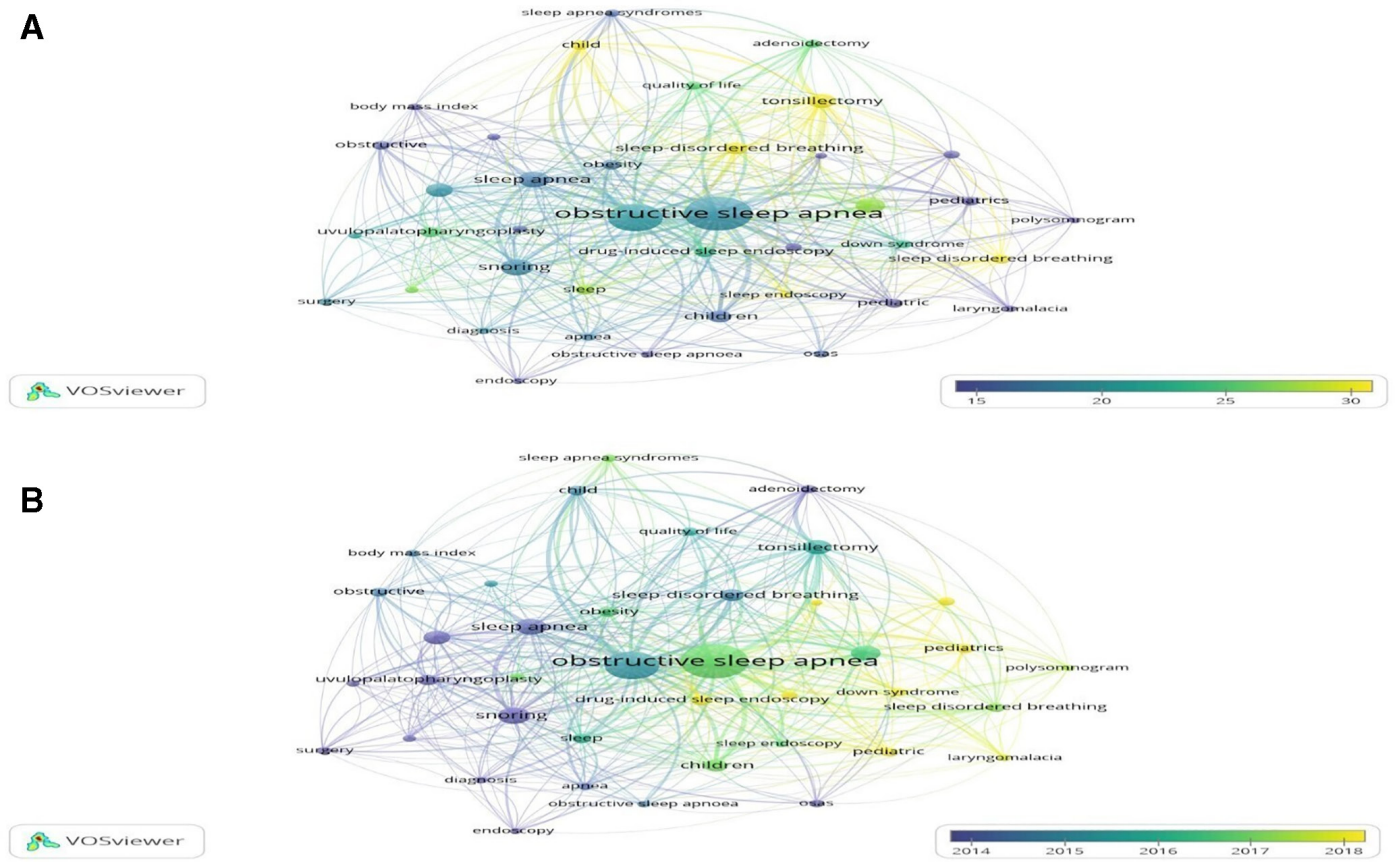
Overlay visualization maps of keyword co-occurrence in polysomnography research. (A) Keywords are colored according to their average citation counts, as indicated by the color scale from blue to yellow, with warmer colors representing more highly cited topics. (B) Temporal overlay visualization showing the evolution of research topics over time. The color scale in the lower right corner represents the average publication year of the documents in which a keyword appears, with blue indicating earlier topics and yellow indicating more recent topics. Node size corresponds to keyword occurrence frequency, and links represent co-occurrence relationships between keywords.

The results of the cluster analysis, illustrating the combinations of keywords, are depicted in Figure 5. Figure 5A depicts a co-occurrence map of the most frequent keywords. Figure 5B depicts temporal trends in keyword mapping, comparing earlier and recent publications.

### 3.4. Cross-database citation comparison

Through a comparative analysis of citation counts across the 4 databases, all sources demonstrated very strong correlations with WoS. OpenAlex showed the highest correlation with WoS (*r* = 0.983, *R*^2^ = 0.966), followed by Crossref (*r* = 0.981, *R*^2^ = 0.962) and Google Scholar (*r* = 0.967, *R*^2^ = 0.935). Spearman correlation coefficients confirmed these findings, with all pairwise comparisons yielding statistically significant, nearly perfect positive associations (ρ = 0.960–0.969, *P* < .001).

**Figure 6.**
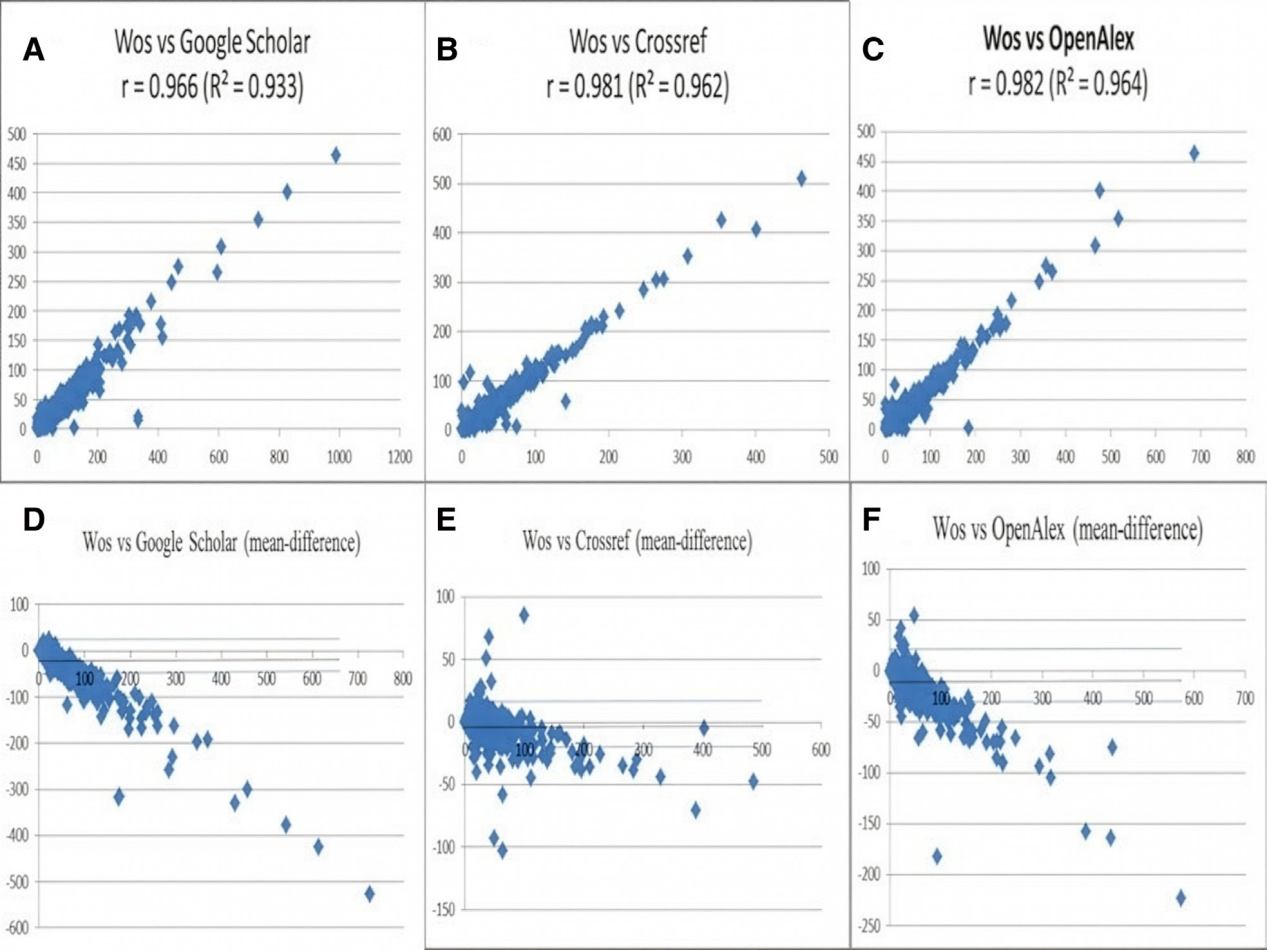
Cross-database citation comparability and bias analyses. (A–C) Scatter plots showing Pearson correlations (*r*) and coefficients of determination (*R*^2^) between absolute citation counts in Web of Science versus Google Scholar (A), Crossref (B), and OpenAlex (C) on linear axes. (D–F) Bland–Altman mean-difference plots illustrating systematic citation bias for WoS versus Google Scholar (D), Crossref (E), and OpenAlex (F). The horizontal black line represents the mean absolute bias, and the horizontal blue lines indicate the 95% limits of agreement (calculated as mean bias ± 1.96 SD). Each data point represents an individual original article. SD = standard deviation, WoS = Web of Science.

Bland–Altman plots revealed a systematic negative bias, indicating that WoS consistently yielded lower citation counts than the other databases. The mean bias was −18.41 citations for WoS versus Google Scholar (95% limits of agreement: −85.47 to +48.64), −7.70 citations for WoS versus OpenAlex (95% limits of agreement: −36.76 to +21.36), and −2.31 citations for WoS versus Crossref (95% limits of agreement: −17.70 to +13.09). While most differences fell within the ±1.96 standard deviation limits of agreement, discrepancies increased for highly cited articles. Figure 6A–F depicts findings via scatter plots and Bland–Altman mean-difference plots with bias lines and ±1.96 standard deviation limits of agreement.

## 4. Discussion

Beyond the field-specific findings, our analysis demonstrated that using multiple databases enhances retrieval coverage. PubMed retrieved additional articles missed by the initial WoS query; most of these could later be found in WoS only through manual search, suggesting that indexing and search algorithms differ across databases. Furthermore, 7 of the 40 most highly cited articles were retrieved exclusively from PubMed, emphasizing the importance of multiple database searches to minimize bias in bibliometric analyses.

Our analysis identified a biphasic publication pattern, with the first stage covering 1995 to 2010 and a second stage covering 2011 to 2025. On average, 34 articles (range: 11–62) were published annually. From 2011 to 2025, article counts showed an upward trend, averaging 86 publications annually (range: 63–113). Our projections for 2026 and beyond suggest a slight increase in PSG research.

Nineteen of the 23 leading countries (≥10 articles) in PSG research were classified as developed economies by the International Monetary Fund,^[10]^ while 4 – Türkiye, China, Brazil, and Egypt – were emerging countries with major economies. Countries with higher gross domestic product and gross domestic product per capita produced more articles, suggesting an association between economic resources and research productivity. Statistical analyses showed that advanced economies published significantly more than developing ones (Mann–Whitney *U*, *P* < .05). These findings align with prior studies and are consistent with the idea that sustained funding, institutional support, and international collaboration are associated with research output.^[11–13]^

The analysis revealed that journals such as the Laryngoscope, Otolaryngology–Head and Neck Surgery, International Journal of Paediatric Otorhinolaryngology, and European Archives of Otorhinolaryngology, among others, played a central role in shaping the global PSG literature. These journals are considered suitable platforms for researchers aiming to publish their work on this subject.

Analysis of the average number of citations per article showed that the leading journals were Archives of Otolaryngology Head and Neck Surgery (56.50), the Laryngoscope (33.28), and American Journal of Rhinology and Allergy (29.27), among others. These journals may be strategic options for authors seeking higher visibility.

Among the articles, Friedman et al^[14]^ conducted a study in the Laryngoscope titled “Clinical predictors of obstructive sleep apnea” with the highest impact according to the total number of citations. The second most-cited study was conducted by Franco et al^[15]^ in Otolaryngology–Head and Neck Surgery titled “Quality of life for children with obstructive sleep apnea.” The third most-cited study was conducted by Friedman et al^[16]^ titled “Clinical staging for sleep-disordered breathing” published in Otolaryngology–Head and Neck Surgery. The fourth and fifth most-cited studies were those of Suen et al^[17]^ and Chervin et al,^[18]^ published in Archives of Otolaryngology Head and Neck Surgery. Notably, 19 of the 20 most-cited studies were published in the Laryngoscope, Archives of Otolaryngology Head and Neck Surgery, and Otolaryngology–Head and Neck Surgery journals, highlighting the substantial influence of these journals on PSG research.

Of all articles, the most impactful study, averaging 21.4 citations annually, was Woodson et al,^[19]^ published in Otolaryngology–Head and Neck Surgery titled “Three-Year Outcomes of Cranial Nerve Stimulation for Obstructive Sleep Apnea: The STAR Trial.” The second most impactful study was Friedman et al^[14]^ titled “Clinical predictors of obstructive sleep apnea.” The third most impactful study was Franco et al^[15]^ titled “Quality of life for children with obstructive sleep apnea.” The fourth most impactful study was Friedman et al^[16]^ titled “Clinical staging for sleep-disordered breathing.” The fifth most influential study was Vroegop et al^[20]^ titled “Drug-Induced Sleep Endoscopy in Sleep-Disordered Breathing: Report on 1249 Cases.”

The co-citation analysis highlighted several high-impact references, including studies by Young et al,^[21]^ Sher et al,^[22]^ Berry et al,^[23]^ Johns,^[24]^ Iber et al,^[25]^ and Marcus et al,^[26]^ among others. These studies serve as essential references for scholars interested in this area.

Six main research themes emerged from the cluster analysis: OSA, airway obstruction, pediatric OSA, drug-induced sleep endoscopy (DISE), complications, and adenotonsillectomy. The most frequently cited keywords over time were OSA, PSG, snoring, sleep apnea, adenotonsillectomy, tonsillectomy, pediatric OSA, sleep-disordered breathing, uvulopalatopharyngoplasty, and DISE.

The keyword analysis revealed 12 keywords that represent emerging trends (Fig. 4). The main research trends observed in recent years were DISE, hypoglossal nerve stimulation, upper airway stimulation, sleep surgery, transoral robotic surgery, surgical treatment of obstruction, sleep medicine, and sleep stages. These trends are consistent with recent developments in PSG treatment.

Until 2014, the primary areas of focus included snoring and surgical treatment options. Since 2016, research themes related to PSG diagnostic methods, DISE, robotic surgery, and upper airway stimulation have become more popular.

Taken together, these findings suggest that PSG research in otorhinolaryngology has remained centered on long-standing clinical themes such as OSA, DISE, surgical management, and associated complications for 3 decades. Although relatively recent topics such as transoral robotic surgery and hypoglossal nerve stimulation have appeared in cluster and trend analyses, they remain underrepresented.

In addition to these bibliometric patterns, a distinctive methodological contribution of the present study is the comparative analysis of citation counts across 4 databases (WoS, Google Scholar, Crossref, and OpenAlex). Although there were very high correlations among them, Bland–Altman analysis revealed substantial systematic differences in absolute citation counts. Compared with WoS, Google Scholar, and OpenAlex tended to report higher citation counts with wider limits of agreement, particularly for highly cited articles, whereas Crossref showed smaller differences and closer agreement with WoS. Despite their strong correlations and suitability for analyzing trends and relative rankings, these databases are not fully equivalent when reporting absolute citation counts, particularly for highly cited articles, where discrepancies become more evident. OpenAlex and Crossref provide the most consistent alternatives to WoS, whereas Google Scholar, despite its broader coverage, should be used cautiously. Therefore, the selection of a database should align with the specific purpose of the bibliometric evaluation: while trend monitoring may tolerate broader sources, performance assessment and policy-making require stricter database consistency.

On the basis of the available literature, no previous bibliometric study has specifically examined PSG research in the field of otorhinolaryngology. Only 3 related studies were identified: Robert et al,^[27]^ Huang,^[5]^ and Pan et al.^[28]^ However, none of these focused on otorhinolaryngology journals. Robert et al’s study is limited by timeframe and broader in scope, covering general sleep science over 40 years (1974–2004). Huang et al analyzed OSA articles between 1991 and 2006 but relied solely on WoS. Pan et al used a similar methodology yet restricted their analysis to OSA across disciplines over 10 years. To our knowledge, this study is the first to focus exclusively on PSG research in otorhinolaryngology journals. It provides a clearer understanding of the clinical and surgical role of PSG. It also offers a comprehensive analysis, strengthened by a cross-database citation comparison, highlighting the methodological impact of database selection on bibliometric outcomes.

Our methodological approach parallels previous bibliometric studies.^[11–13]^ However, our study goes further by comparing citation counts across 4 databases and cross-checking search results through PubMed to capture articles that were not initially retrieved in WoS.

In our study, we limited our search to WoS and PubMed for article retrieval and selected 4 databases for citation analysis. A potential limitation is the exclusion of Scopus, another major bibliometric database that may introduce coverage bias. Although Scopus was not included in the retrieval stage, this decision was intentional rather than arbitrary. Previous studies have shown that database selection directly affects bibliometric assessments. WoS emphasizes selectivity and detailed categorization, whereas Scopus covers a larger number of journals but spans a shorter historical period. Notably, WoS and Scopus differ substantially in terms of journal coverage and subject classification logic; however, they show high agreement in biomedical fields, supporting the adequacy of single-database approaches for specialty-specific analyses.^[29,30]^ Since this study aimed to evaluate PSG research specifically within the predefined WoS otorhinolaryngology category rather than compare database coverage, WoS was the preferred source. Future bibliometric studies incorporating Scopus and other emerging databases could broaden journal coverage and enable more detailed cross-platform comparisons, thereby providing further insights into the influence of database selection on bibliometric outcomes.

## 5. Conclusion

This study mapped 3 decades of PSG research in otorhinolaryngology, demonstrating a steady increase in output with recent emphasis on DISE, upper airway stimulation, sleep medicine, and robotic surgery. It also showed that citation counts vary across databases and should not be used interchangeably for absolute evaluation. Thus, this study offers a field-specific overview of research trajectories and methodological insights into the impact of database selection.

## Author contributions

**Conceptualization:** Ömer Faruk Zengin, Gözde Salihoglu Zengin.

**Data curation:** Ömer Faruk Zengin, Gözde Salihoglu Zengin.

**Formal analysis:** Ömer Faruk Zengin, Gözde Salihoglu Zengin.

**Methodology:** Ömer Faruk Zengin, Gözde Salihoglu Zengin.

**Supervision:** Ömer Faruk Zengin, Gözde Salihoglu Zengin.

**Writing – original draft:** Ömer Faruk Zengin.

**Writing – review & editing:** Ömer Faruk Zengin, Gözde Salihoglu Zengin.
